# Study of the Motivation of Spanish Amateur Runners Based on Training Patterns and Gender

**DOI:** 10.3390/ijerph17218185

**Published:** 2020-11-05

**Authors:** David Manzano-Sánchez, Lucas Postigo-Pérez, Manuel Gómez-López, Alfonso Valero-Valenzuela

**Affiliations:** 1 Department of Physical Activity and Sport, Faculty of Sport Sciences, University of Murcia, Santiago de la Ribera, 30720 Murcia, Spain; david.manzano@um.es (D.M.-S.); avalero@um.es (A.V.-V.); 2 Faculty of Sport Science, University of Murcia, Santiago de la Ribera, 30720 Murcia, Spain; lucas.postigop@um.es

**Keywords:** athletics, perception of success, sport events, motives, achievement goals

## Abstract

The objectives of the present study are to analyze the different training patterns of the amateur runners, according to their gender, and to find out a correlation between the training pattern and the motivation. The sample was composed of 457 amateur runners. For the collection of data, a two-part questionnaire was used. The first part consisted of questions about sporting and healthy patterns and the second part consisted of the Perception of Success Questionnaire (POSQ), adapted to Spanish. The obtained results indicated that their motives for starting to practice running and to continue their involvement are health and fun. The training pattern is as follows: they practise one to three days per week, running from three to five hours overall plus additional stretching and high intensity training. They participated in less than one running event per month. Most of them did not belong to an athletic club, did not have a coach, were not federated and have more than four years’ experience of running. What concerns the gender differences, the men trained more than the women, and they did it with relatives and friends; women preferred to do it with friends or by themselves with the assistance of a coach. Age and running hours per week were the best variables to predict the task goal orientation, especially for men. For women, training hours per week predicted the goal orientation but to the ego. This finding could be especially helpful for coaches. A high number of training hours for men was linked with a task goal orientation, and on the other hand, for women it meant an ego goal orientation. The consequences of their behaviours were likely to be markedly different.

## 1. Introduction

Over the last decades there has been a “boom” in athletic events at the international level, and it is increasingly common for the adult population to participate in amateur races [1]. This phenomenon can be seen in Spain [2]. It emerged in the 1990s and was influenced by postmodernism and the celebration of the Barcelona 1992 Olympic Games [3].

The number of studies that have discussed the characteristics of amateur runners who participate at a local, national, or even international level has increased over the past few years. Some of the main themes have been related to physical health benefits [4,5], psychological benefits [6], tourism and leisure [7,8], and the motivational characteristics of amateur runners [9,10].

Psychological studies have focused on why people begin and continuing to practise athletics [11,12,13], and also why some take part in amateur races [14,15]. One of the most recent studies [16] identified health as the main reason for participation in long-distance races, followed by personal growth and greater self-esteem. Such factors accord with previous research, but not necessarily in that order of importance [14,15]. In another study [17], long-distance runners stated that they were driven by the desire to experience strong emotions, have fun, and to enjoy the atmosphere of the sporting event. 

The goal orientation of athletes helps towards an understanding of the reasons why amateur runners participate in races [18], and why it is so important for them to be involved [19]. Many of these can be explained using the achievement goal theory [20]. This states that ego goal orientation is related to the belief that success in sport is achieved by being more skilled than the opponent, even if this means using techniques of deception. In contrast, task orientation is related to the most adaptive motivational patterns, which consist in believing that success in sport is achieved through personal effort. In this case, sport is about training and individual development. In this sense, the task-oriented athlete allows to feel satisfaction with the sport practise in both genders [21].

Previous studies have related goal orientation to sociodemographic variables, especially gender and age. They have indicated that the older the participant, the greater his or her task goal orientation, and conversely, the younger the participant, the greater his or her ego orientation [22,23,24]. On the other hand, for the gender variable, the results are somewhat contradictory, since despite the fact that there are studies showing that motives are more linked to a profile focused on performance and competition for men tan for women [23,25,26], the studies that have analysed goal orientation have not found significant differences between the genders [27,28,29].

Another important aspect to consider when studying the goal orientations of amateur athletes is their training experience and patterns, advice from a coach, partners with whom they train, training time and days per week, and other variables [30]. Other aspects include variables age, level of studies, sports habits, and running addiction contributed to differentiating the motives to practise running [31]. These differences between genders justify the importance to create a profile of the amateur runner [32].

Using the data provided, the aim of the present study is to discover the training patterns of long-distance runners in the Murcia region of Spain (races from 5 to 14 kilometres), to analyse the reasons why they begin and continue to participate in the sport, and to examine the relationship between their training patterns and goal orientation, based on gender difference.

## 2. Materials and Methods

### 2.1. Design and Participants

This is a descriptive, quantitative, cross-sectional study. The sample was chosen randomly from amongst participants in two amateur races in Murcia, both of which are part of the provincial road racing circuit of the region. Both races had two distances, 5 k and 10 k and 7 k and 14 k, respectively. In the first race there were almost 200 runners in total and in the second almost 3000. Participants filled out the questionnaire before the race, specifically when they picked up their numbers. All of them provided written informed consent to participation in the survey. After reviewing and entering the questionnaires into a database, 14 were eliminated because the respondents had filled them out incorrectly, had omitted essential data (such as age or gender), or both. Once these had been eliminated, 486 participants remained. However, 29 additional participants also had to be excluded after abnormal values were obtained in the data cleaning. This left 457 subjects.

Then, the data exclusion criteria were applied. First, debugging was performed to detect atypical cases. This was followed by a reliability analysis using Cronbach’s alpha. Finally, normality was calculated using the Kolmogorov−Smirnov test. Of the participants, 350 were men and 107 were women. The age range was 18 to 63 years and the mean age was 37.84 ± 9.139 years. The mean age for the men was 38.23 ± 9.346 years and 36.56 ± 8.34 years for the women.

### 2.2. Measurement Instruments

The questionnaire consisted of 34 items divided into two sections. The first comprised a form designed to collect demographic and training information. Participants answered questions on the following variables: age, gender, athletic club, federation, sporting experience, reasons for starting and maintaining participation in amateur races, planning of training sessions, number of hours and days of training per week, days of high intensity training per week, the number of runners the participant typically associated with during a week, days of strength training per week, and best finish time in 5 k, 10 k, or half marathon races. Most of the questions were extracted from Ogles and Masters [20].

The second section comprised questions from the Spanish version of the Escartí, Cervelló and Guzmán Perception of Success Questionnaire [33]. These were extracted from the Perception of Success Questionnaire (POSQ) by Roberts and Balagué [34]. This questionnaire is based on the achievement goals theory and consists of twelve items, six of which measure ego or performance orientation and the other six task or mastery orientation. The answers in this questionnaire are Likert-type, ranging from (1) totally disagree to (4) totally agree. The reliability of the instrument was verified through internal consistency analysis. Applying Cronbach’s alpha, values of 0.770 were obtained on the ego orientation scale and 0.737 on the task orientation scale. Approval for the study was obtained from the Ethics Committee of Murcia University, Spain. The file number was 2266/2019. The study was consistent with the Helsinki declaration of 1975. The goodness of the fit indices (RMSEA = 0.023; CFI = 0.997; TLI = 0.995) of the scale in the study sample was checked by the confirmatory factor analysis (CFA) denoting a good fit to the data. Two additional measurements were taken into account to evaluate the reliability of the scale: the composite reliability (CR) with a value of 0.783 and 0.743 for ego and task factor respectively, higher than the 0.70 value recommended by the literature [35], and the average variance extracted (AVE) with a value of 0.553 for ego and 0.496 for task, this second one very close to 0.50, value recommended [36]. Additionally, the convergent validity was verified through the values of the t test associated with the factorial loads of the items, which were higher than 1.96 (*p* < 0.05). Furthermore, the discriminant validity, which has to do with seeing the clear distinction between any pair of constructs, was evaluated using the method suggested by Fornell and Larcker [37]. This method demonstrates discriminant validity if the square root of AVE value of a determined factor, in this case 0.305 for ego and 0.246 for task were greater than the correlation coefficients between the determined factor and any other factor in the proposed scale (0.208). 

### 2.3. Procedure

Before completing the questionnaire, the participants were given an information sheet and were asked to sign an informed consent form. Before the “Save the Children” race took place, the organisers were contacted to explain the objectives of the study, and a model of the questionnaire was delivered to them. For the race at the Air Base in Alcantarilla, we contacted the Murcian Athletics Federation, who then contacted the Military Academy (the organisers). After a short period of time, a favourable response was received. Data collection was carried out before the contest, the day before the races, and when the runners picked up their numbers. Participants were informed of the purpose of the research, and were told that it was voluntary and confidential. All subjects had to be of legal age. They were given the opportunity to raise any doubts they may have had with regard to the study.

### 2.4. Data Analysis

Data analysis was carried out using the software package SPSS version 22.0 (SPSS Inc. Chicago, IL, USA) and Mplus version 8.4. First, the chi-square test for normality was performed for the categorical variables; a non-normal distribution (*p* < 0.05) was obtained. Then, normality was performed for the continuous variables through the Kolmogorov-Smirnov test; a non-normal distribution (*p* < 0.05) was obtained. For the analysis of the categorical variables, the chi-square statistic was used for contingency tables (analysing the differences according to gender), and the contingency coefficient was performed to show the correlation between the variables. An analysis of continuous variables was carried out using the Mann−Whitney U test to contrast them with gender, and a correlation analysis was carried out using Kendal’s Tau b test to verify the relationship between variables related to training patterns with the age and motivational orientation within the 95% confidence interval was also carried out. Finally, three multivariate linear regression models were used to verify the prediction of the variables of training patterns on motivational orientation to ego and task, on the global sample, and as a function of gender.

## 3. Results

### 3.1. Descriptive Analysis of Reasons for Starting and Continuing Sports Practice

The main reason for participating in the races selected for the study was to have fun (30.9%), followed by health and to have fun (17.7%). The main reason for continuing to participate in amateur races was to have fun (26.5%), followed by health and to have fun (19.3%), then health, relationships, and to have fun (12.3%).

**Figure 1:**
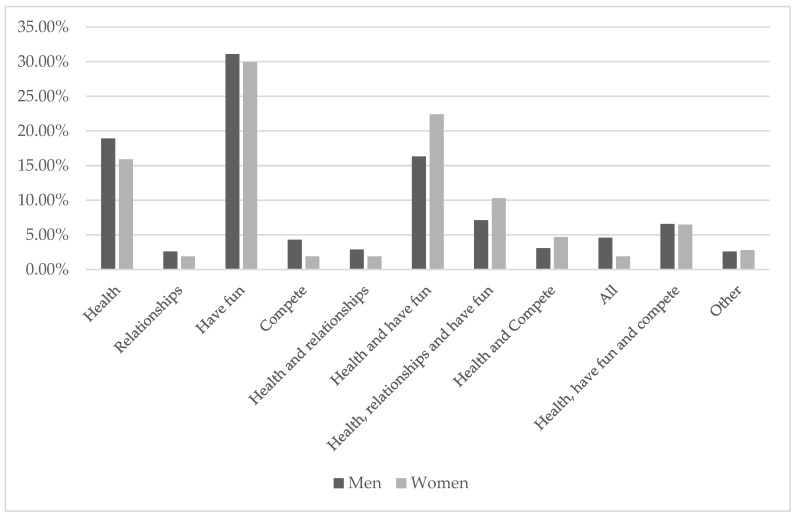
Reasons for starting sports according to the gender of the participants.

**Figure 2:**
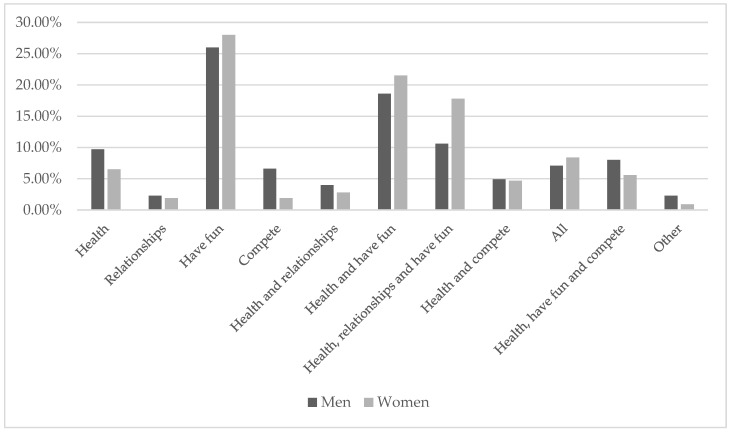
Reasons for continuing sports according to the gender of the participants.

With regard to gender (Figure 1 and Figure 2), we observed the same trend; men gave a greater number of reasons for why they started and continued to compete than women, who gave more combined reasons (health and to have fun or health, relationships, and to have fun). In both cases, the predominant motive was to have fun (31.1% and 29.9% initially and 26% and 28% thereafter).

### 3.2. Differences in Training Patterns in the Total Sample and Their Relationship with the Gender of the Participants

An analysis was carried out, taking into account the training patterns of the athletes and distinguishing them according to the gender of the participants (Table 1).

Statistically significant differences were obtained at *p* ≤ 0.001 in all variables except the practise of other sports. The total sample obtained statistically significant differences in *p* = 0.008 (56.2% of the sample) in favour of the group that did not belong to a club and in *p* = 0.004 for flexibility training days (31.7% in favour of those who trained one day a week). Statistically significant differences were found in favour of the non-federated group (*p* ≤ 0.001, 73.7%), who had more years of experience (in favour of the group with more than four years at *p* ≤ 0.001, 50.8%) and who carried out training planning on their own (*p* ≤ 0.001, 69.1%). There are differences (*p* ≤ 0.001) between goal orientation, with a value of 3.53 on task orientation and 1.91 on ego orientation.

With regard to training patterns, in addition to the flexibility training mentioned above, the greater number of respondents ran one to three days a week (49%), and did one day (32.4%) or two (37.4%) of high intensity training. Sessions were predominantly with friends and family (35.4%). The greater percentage of participants ran between three to five hours per week (53.6%), and 72.2% of the sample did strength training. A number of participants usually dedicated two days to strength training (32.2%; 27.8% did none), mainly in the gym (36.9%). Finally, the performance of other sports and health considerations were related to the frequency of competition. There were statistically significant differences in the frequency of competition in the sample; 41.8% competed less than once a month.

Statistically significant differences were obtained in the years of experience, with men having more years of experience compared to women (*p* = 0.002). Differences were also found in running hours per week (*p* = 0.008) with men training more hours than women, in addition to the training partner variable (*p* = 0.004), where men trained more with friends and family and women more alone and with friends. Finally, the difference between men’s involvement in other sports and women’s was statistically significant (*p* ≤ 0.001).

### 3.3. Correlation Analysis According to Motivational Orientation and Sociodemographic Variables

As a preliminary step to the regression analysis and the identification of possible training variables that predict the motivational orientation of amateur runners, a correlation analysis was carried out to see the relationship between the variables of training patterns of the sample and the motivational orientation (Table 2).

The results indicate that there is a positive relationship between the running hours they train per week and dispositional orientation towards the task (T = 0.112, *p* < 0.05). On the contrary, there is an indirect correlation between age and ego orientation (T = −0.091, *p* < 0.05). In addition, age was correlated directly with experience (T = 0.277, *p* < 0.05), and indirectly with high intensity training days (T = −0.118, *p* < 0.05) and strength training days.

### 3.4. Regression Analysis According to Motivational Orientation

Finally, in Table 3, following the tests carried out in previous studies [19], a multivariate linear regression analysis was carried out to explain most of the variance, the dependent variables being ego and task scores, using as selection variable gender. The predictor variables were selected based on those that had yielded significant differences in the Mann Whitney U test based on gender, specifically, age, sports experience, running hours per week, performance in other sports, and participation alone or with training partners.

The R values were extracted to explain the variance, Beta the prediction, and F to see the relationship between the variables and their significance. The models obtained were consistent in explaining part of the variance and obtaining some significant relationships. The ego orientation model indicated that it could be significantly predicted for the total sample with task orientation (*p* ≤ 0.001) and age (*p* = 0.020, variance 3.6%): in the case of men, high task orientation (*p* = 0.040, variance of 2.7%) and for the women, age (*p* = 0.012) and running hours per week (*p* = 0.016, variance 16.7%). With regard to task orientation, taking into account the total sample, the model indicated that it could be predicted by ego orientation (*p* = 0.008) and running hours per week (*p* = 0.006), with 4.3% of the total variance. More specifically, the model when applied to the men indicated that the running hours per week (*p* = 0.034) were, together with the ego (*p* = 0.040), the main predictors (variance = 4.7%), whereas there was no meaningful variable for women.

## 4. Discussion

The objective of this study was to learn about the training patterns of long-distance runners in the Region of Murcia, analysing the reasons for starting and continuing to compete, investigating the relationship between their training patterns and motivational orientation, and examining the differences based on gender. 

For both men and women, the first reason for starting and continuing to compete in amateur races was to have fun. The second was health. This was generally in line with previous studies [15,16,17], though the fun factor was only mentioned in the present case. However, in another study of Escartí, Cervelló and Guzmán [33], the athletes had very high scores in fun. In the present study, high values for fun were obtained either of itself or in combination with dimensions such as health.

Most of the subjects trained one to three days a week, and prepared between three to five hours for the race, and there was some variation in sessions and in strength training. They did some high intensity training and participated in less than one competition a month. Parra-Camacho, Alonso Dos Santos, and González-Serrano [31] found that veteran amateur athletes usually trained five days a week and two hours a day. The proportion of time dedicated to running was similar, although in the study by Ruiz-Juan and Zarauz-Sancho [30], the number of sessions per week was higher, which may have been due to differences in the ages of the sample. In the study of Salas-Sánchez, Román, Soto, Santos, and García-Pinillos [32], most of the veteran athletes had four to twelve years’ experience of amateur races, did four training sessions a week, did not have a coach, were not federated, and participated in 10–11 amateur races per year. This is largely in keeping with the present study, which provides new data on the patterns of amateur runners. Here, a higher percentage did not belong to a club, were not federated, and did not have a coach. 

The data from the present study showed that men trained for more hours than women. The studies by [28,32] found the same. However, in these and other studies [27,28,32], the women were less inclined to train alone than men. This contrasts with the findings of the present study, where women tended to train alone or with friends, while men preferred to train with friends or family. The men considered themselves to be more self-sufficient than the women, preferring not to train alone and to do so without the advice of a coach. This was similar to the results of other studies [27,28] where a higher percentage of women than men had coaches.

With regard to motivational orientation, in the present study low scores were obtained in ego orientation and high scores in task orientation. Most of the subjects assigned a higher value to the task, and the main reason for participating in sport was to have fun. These findings are in accordance with Ruiz-Juan, Gómez, Pappous, Alacid and Flores [38] with young elite paddlers and Abraldes, Granero-Gallegos, Baena-Extremera, Gómez-López, and Rodríguez-Suárez [39] with swimmers and Abraldes, Gómez-López, Granero-Gallegos, and Rodríguez-Suárez [40] with lifesavers where it was concluded that task orientation was positively related to enjoyment, and finally with Roberts, Treasure and Balagué [34], where, amongst veterans, higher scores were recorded for fun and intrinsic motivation. Furthermore, an examination of the correlations in the present study indicated that as ages advanced, ego orientation decreased but task orientation remained high; therefore, older athletes were less interested in performance. This is in line with Nikolaidis, Chalabaev, Rosemann, and Knechtle [26]. It indicates that the participants were more motivated by personal reasons (health and personal goal achievement) than by competition or social recognition, and the scores for these were higher the greater the age.

Certain variables related to training patterns can predict the orientation goal of amateur athletes, Zarauz-Sancho and Ruiz-Juan [29] concluded that having a coach can be a predictor of lower task orientation in men and women. In the present study, although this and other related variables were considered, there were no significant differences in terms of gender. However, running hours per week were a good predictor of task orientation for the men. For the women, training hours per week (in line with running hours) was the training pattern variable that most predicted goal orientation (i.e., towards the ego or performance). This leads us to suggest that training patterns had different orientations depending on gender. This might be taken into account when setting goals.

The present study had a number of limitations. For example, the numbers of women and men were not equal, and the sample size was relatively small. The topic is under-researched both in Spain and other countries, so it was difficult to find similar studies. Controlling for the possibly that participants might fill out the questionnaire twice was problematic. Finally, the lack of a standardised and validated questionnaire means that the results of the present study cannot be compared with those of other researchers; one could be developed for similar future projects. 

## 5. Conclusions

The long-distance runners who participated in the present study began and continued to compete in amateur races for reasons of fun or fun combined with health. They trained 1–3 days a week, dedicating 3 to 5 h to the race itself. Most of the study participants did not belong to a club, did not have a coach, and were not federated. They did a certain amount of high intensity, strength, and flexibility training and competed less than once a month. The men trained for longer than the women, and they tended to train either alone or with friends or family. The women were more likely to use the advice of a coach.

The motivation of these amateur runners was high in task and low in ego orientation, which was in keeping with the reasons (the chief one of which was to have fun) that led them to participate in amateur races. Running hours per week and age were the variables that best predicted motivational task orientation, especially in the case of the men. For the women, hours of training per week predicted motivational orientation, but towards the ego or performance. This discovery might be especially valuable for coaches, since the hours of training and hours of running pointed to either a more task-oriented or a more ego-oriented approach depending on the gender of the participants. The participants’ goals were either focused on personal and mastery achievement or on competition and external achievement.

## Figures and Tables

**Table 1:** Data analysis related to the variables of training patterns.

Variable		Men		Women			Total		
		M	%	M	%	*p*/C	M	%	*p*
Federated	Yes	98	28.0%	22	20.6%	0.134	120	26.3%	
	No	252	72.0%	85	79.4%	0.071	337	73.7%	<0.001 ***
Belong to a club	Yes	162	46.3%	38	35.5%	0.058	200	43.8%	
	No	188	53.7%	69	64.5%	0.092	257	56.2%	0.008 **
Experience	Less than 1 year	54	15.4%	28	26.2%		82	17.9%	
	Between 1 and 2 years	39	11.1%	21	19.6%		60	13.1%	
	Between 3 and 4 years	65	18.6%	18	16.8%		83	18.2%	
	More than 4 years	192	54.9%	40	37.4%	0.002 **	232	50.8%	<0.001 ***
						0.071			
Training planning	By myself	250	71.4%	66	61.7%		316	69.1%	
	A coach	70	20.0%	29	27.1%		99	21.7%	
	A friend	14	4.0%	8	7.5%		22	4.8%	
	Other	1	0.3%	1	0.9%		2	0.4%	
	By myself andwith friends	15	4.3%	3	2.8%	0.175	18	3.9%	<0.001 ***
						0.117			
Training days per week	1–3 days	164	46.9%	60	56.1%		224	49.0%	
	4–5 days	140	40.0%	35	32.7%		175	38.3%	
	More than 5 days	46	13.1%	12	11.2%	0.247	58	12.7%	<0.001 ***
						0.078			
High intensity training days per week	0 days	49	14.0%	20	18.7%		69	15.1%	
	1 days	117	33.4%	31	29.0%		148	32.4%	
	2 days	130	37.1%	41	38.3%		171	37.4%	
	More than 2 days	54	15.4%	15	14.0%	0.617	69	15.1%	<0.001 ***
						0.063			
Training partner	Family and friends	138	39.4%	24	22.4%		162	35.4%	
	With friends	79	22.6%	29	27.1%		108	23.6%	
	Clubmates	35	10.0%	10	9.3%		45	9.8%	
	With family	15	4.3%	6	5.6%		21	4.6%	
	Alone and with friends	48	13.7%	25	23.4%		73	16.0%	
	Alone, with friends and clubmates	29	8.3%	11	10.3%		40	8.8%	
	Other	6	1.7%	2	1.9%	0.045 *	8	1.8%	<0.001 ***
						0.166			
Running hours per week	Less than 3 h	75	21.4%	38	35.5%		113	24.7%	
	Between 3 and 5 h	192	54.9%	53	49.5%		245	53.6%	
	More than 5 h	83	23.7%	16	15.0%	0.008 **	99	21.7%	
									<0.001 ***
						0.146			
Strength training	Yes	253	72.3%	77	72.0%		330	72.2%	
	No	97	27.7%	30	28.0%	0.948	127	27.8%	<0.001 ***
						0.003			
Strength training days	0 days	98	28.0%	29	27.1%		127	27.8%	
	1 day	91	26.0%	23	21.5%		114	24.9%	
	2 days	111	31.7%	36	33.6%		147	32.2%	
	More than 2 days	50	14.3%	19	17.8%	0.696	69	15.1%	
						0.056			<0.001 ***
Type of strength training	Gym	89	35.2%	33	42.3%		122	36.9%	
	Hills	41	16.2%	6	7.7%		47	14.2%	
	Body circuits	34	13.4%	8	10.3%		42	12.7%	
	Other	2	0.8%	4	5.1%		6	1.8%	
	Slopes and body circuits	31	12.3%	12	15.4%		43	13.0%	
	All the above	56	22.1%	15	19.2%	0.080	71	21.5%	<0.001 ***
						0.155			
Flexibility training days	0 days	81	23.1%	18	16.8%		99	21.7%	
	1 day	110	31.4%	35	32.7%		145	31.7%	
	2 days	66	18.9%	30	28.0%		96	21.0%	
	More than 2 days	93	26.6%	24	22.4%	0.145	117	25.6%	
						0.108			0.004 **
Oher sports	Yes	188	53.7%	48	44.9%	0.109	236	51.6%	
	No	162	46.3%	59	55.1%	0.075	221	48.4%	0.483
Competition frequency	More than 1 per month	100	28.6%	22	20.6%		122	26.7%	
	1 per month	111	31.7%	33	30.8%		144	31.5%	
	Less than 1 per month	139	39.7%	52	48.6%	0.171	191	41.8%	
									<0.001 ***
						0.088			
		M	SD	M	SD	*p*	M	SD	*p*
Goal orientation	Ego	1.90	0.85	1.91	0.89	0.925	1.91	0.86	
	Task	3.55	0.68	3.48	0.69	0.382	3.53	0.68	<0.001 ***

Note: *p* = * < 0.05, ** < 0.01, *** < 0.001; M = Mean; SD = Standard deviation; Contingency coefficient.

**Table 2:** Correlations between variables related to training patterns and motivational orientation.

Variable	2	3	4	5	6	7	8	9	10
1 Experience	0.208 **	0.121 **	0.258 **	−0.021	0.024	−0.195 **	0.277 **	−0.047	−0.015
2 Training days per week	-	0.475 **	0.47 6 **	0.208 **	0.138 **	−0.245 **	−0.008	−0.014	0.047
3 High intensity training days per week	-	-	0.309 **	0.335 **	0.153 **	−0.184 **	−0.118 **	−0.030	0.016
4 Running hours per week	-	-	-	0.046	0.042	−0.280 **	0.061	−0.007	0.112 **
5 Strength training days	-	-	-	-	0.254 **	0.023	−0.126 **	−0.025	0.011
6 Flexibility training days	-	-	-	-	-	0.008	0.021	0.010	−0.010
7 Competition frequency	-	-	-	-	-	-	−0.081 *	0.009	−0.021
8 Age	-	-	-	-	-	-	-	−0.091 **	−0.040
9 Ego	-	-	-	-	-	-	-	-	0.066
10 Task									

Note: *p* = * < 0.05, ** < 0.01, *** < 0.001.

**Table 3:** Multivariate linear regressive analysis according to the perception of the ego-task and the gender of participants in relation to training patterns (men *n* = 350, women *n* = 107).

Variable	Ego						Task					
	Men		Women		Total		Men		Women		Total	
	Beta	Sign.	Beta	Sign.	Beta	Sign.	Beta	Sign.	Beta	Sign.	Beta	Sign.
Ego	---	---	---	---	---	---	0.110	0.040 *	0.189	0.068	0.124	0.008 **
Task	0.112	0.040 *	0.174	0.068	0.125	0.000 ***	---	---	---	---	---	---
Age	−0.085	0.142	−0.250	0.012 *	-0.117	0.020*	−0.104	0.070	0.064	0.544	−0.066	0.189
Experience	−0.008	0.893	−0.089	0.408	-0.018	0.724	−0.029	0.622	−0.134	0.235	−0.051	0.321
Running hours per week	−0.062	0.268	0.248	0.016 *	0.006	0.911	0.117	0.034 *	0.150	0.167	0.135	0.006 **
Other sports	−0.021	0.706	−0.118	0.218	-0.034	0.474	−0.034	0.524	0.077	0.443	-0.014	0.773
Sportmates	0.019	0.729	−0.037	0.692	0.008	0.865	−0.090	0.090	0.144	0.140	−0.40	0.385
	R^2^ = 0.027 F = 1.557		R^2^ = 0.167 F = 3.336		R^2^ = 0.036 F = 2.793		R^2^ = 0.047 F = 2.810		R^2^ = 0.094 F = 1.719		R^2^ = 0.043 F = 3.346	

Note: *p* = * < 0.05, ** < 0.01, *** < 0.001.
